# Livestock health and disease economics: a scoping review of selected literature

**DOI:** 10.3389/fvets.2023.1168649

**Published:** 2023-09-19

**Authors:** Alexander Kappes, Takesure Tozooneyi, Golam Shakil, Ashley F. Railey, K. Marie McIntyre, Dianne E. Mayberry, Jonathan Rushton, Dustin L. Pendell, Thomas L. Marsh

**Affiliations:** ^1^The Lewin Group, Falls Church, VA, United States; ^2^School of Economic Sciences and Paul G. Allen School for Global Health, Washington State University, Pullman, WA, United States; ^3^Department of Agricultural Economics, Kansas State University, Manhattan, KS, United States; ^4^Department of Sociology, Oklahoma State University, Stillwater, OK, United States; ^5^Modelling, Evidence and Policy Group, School of Natural and Environmental Sciences, Newcastle University, Newcastle upon Tyne, United Kingdom; ^6^CSIRO Agriculture and Food, Saint Lucia, QLD, Australia; ^7^Institution of Infection and Global Health, University of Liverpool, Liverpool, United Kingdom

**Keywords:** animal health economics, global burden of animal disease, livestock production, consumer demand, trade and regulations

## Abstract

Animal diseases in production and subsistence environments have the potential to negatively affect consumers, producers, and economies as a whole. A growing global demand for animal sourced food requires safe and efficient production systems. Understanding the burden of animal disease and the distribution of burden throughout a value chain informs policy that promotes safe consumption and efficient markets, as well as providing more effective pathways for investment. This paper surveys existing knowledge on the burden of animal disease across economic categories of production, prevention and treatment, animal welfare, and trade and regulation. Our scoping review covers 192 papers across peer-reviewed journals and reports published by organizations. We find there exists a gap in knowledge in evaluating what the global burdens of animal diseases are and how these burdens are distributed in value chains. We also point to a need for creating an analytical framework based on established methods that guides future evaluation of animal disease burden, which will provide improved access to information on animal health impacts.

## Introduction

Livestock products represent almost half the value of agricultural production worldwide (1). The production of livestock serves to address consumer demand for animal sourced foods, non-food items (e.g., hides), production inputs (e.g., fertilizer for crops), as well as for other, non-market purposes (e.g., culture). Due to an increasing population and growth in incomes spurred by economic development in rural areas (2–4), it is estimated that by 2050, global demand for meat and milk products may increase by 63 and 30%, respectively (5, 6). Assuming no changes in *per capita* consumption, the average demand for total animal source foods will increase from 1.4 billion to 2.0 billion tons by 2050 (7). Within developing countries, livestock milk and meat production has moved from accounting for 31 and 22% of global meat and milk production, respectively, to 63 and 53% of the same respective global production over the time period 1973–2013 (8). The value of livestock product share in agriculture will continue to increase because of continued growth in demand for animal sourced food products resulting from real income and population growth (9–11).

Livestock disease externalities negatively impact production and distort values due to domestic and international market shocks resulting in market inefficiencies (12). Herd health and sustainability of commercialized livestock product markets, as well as their growth to support demand, and smallholder farming systems are threatened by livestock disease outbreaks and occurrences in production (1). Livestock diseases can also encourage unsustainable and damaging practices. For example, antibiotics to promote growth may be used to increase animal size or address persistent infections, but the overuse of antibiotics may then contribute to adverse societal impacts, such as antimicrobial resistance. Episodic, or unpredictable disease outbreaks similarly reduce animal production but may also have unintended consequences on demand and supply of other market goods. Negative information and publicity tied to a disease outbreak can distort consumer demand in retail markets, while distortions in non-allied markets can lead to shortages or surplus (13).

Livestock disease and/or its externalities can directly affect the health of human and wildlife populations or be affected by climate change or the environment. This can occur through impacts on local environments surrounding livestock production systems (14, 15). For instance, there could be livestock-wildlife disease vector feedback loops (16, 17). The expansion of humans into wildlife areas for urban development and/or livestock production provides a greater opportunity for zoonotic interaction between wildlife-livestock vectors. Changes in climatic conditions have also promoted reemergence of zoonotic pathogens (18) and create production environments with greater burden of livestock disease (19), calling attention to the livestock-wildlife-climate interaction and pathways of disease and subsequent burden. While we acknowledge that the livestock-wildlife interaction is complex and important, we do not do it full justice in this review, leaving it for future efforts.

The importance of livestock disease in production and its effect on the health of populations and markets is reflected in the need and use of government response and eradication programs, as well as the need and use of trade bans and trade restrictions. Previous research has identified and called attention to cost–benefit analysis of disease response, eradication, and detection programs (20, 21), to the impacts of disease on productivity, value, and costs at both production and consumption levels (22–24), the impacts on trade (25, 26), and the externalities and producer decision-making processes associated with disease prevention and treatment (27, 28).

In response to these advancements, our primary objective of this paper is to provide a scoping literature review of livestock health and disease economics to better understand the state of knowledge and identify gaps in defining the burden of animal disease. The following research questions were investigated: (1) What is the economic impact/cost of a given disease on production, prevention and treatment, animal welfare, and trade and regulation? (2) How is this burden apportioned among consumers, producers, and the government? (3) How are climate change, zoonosis, and animal welfare affecting the dynamics of animal health systems? By better understanding the current knowledge and finding animal disease knowledge gaps, this will further support the need for a systematic framework for future evaluation of the burden of animal diseases on producers, consumers, markets and trade, and secondary industries.

Our approach revolves around key principles in animal health economics: livestock production, consumer demand, and trade and regulations. This review begins with livestock production and disease externalities, weaving in issues of climate change and zoonosis. Animal disease prevention and treatment is covered next. Considerable space is allocated for animal welfare, including consumer preferences, as it is important and recommended as a gap in the literature by Hennessy and Marsh (13). Trade and regulation impacts are presented to address global interconnectivity of markets and the role of commerce. We conclude with a discussion of some remaining gaps in existing literature, pressing needs, and opportunities for future research.

## Methods

**Figure 1:**
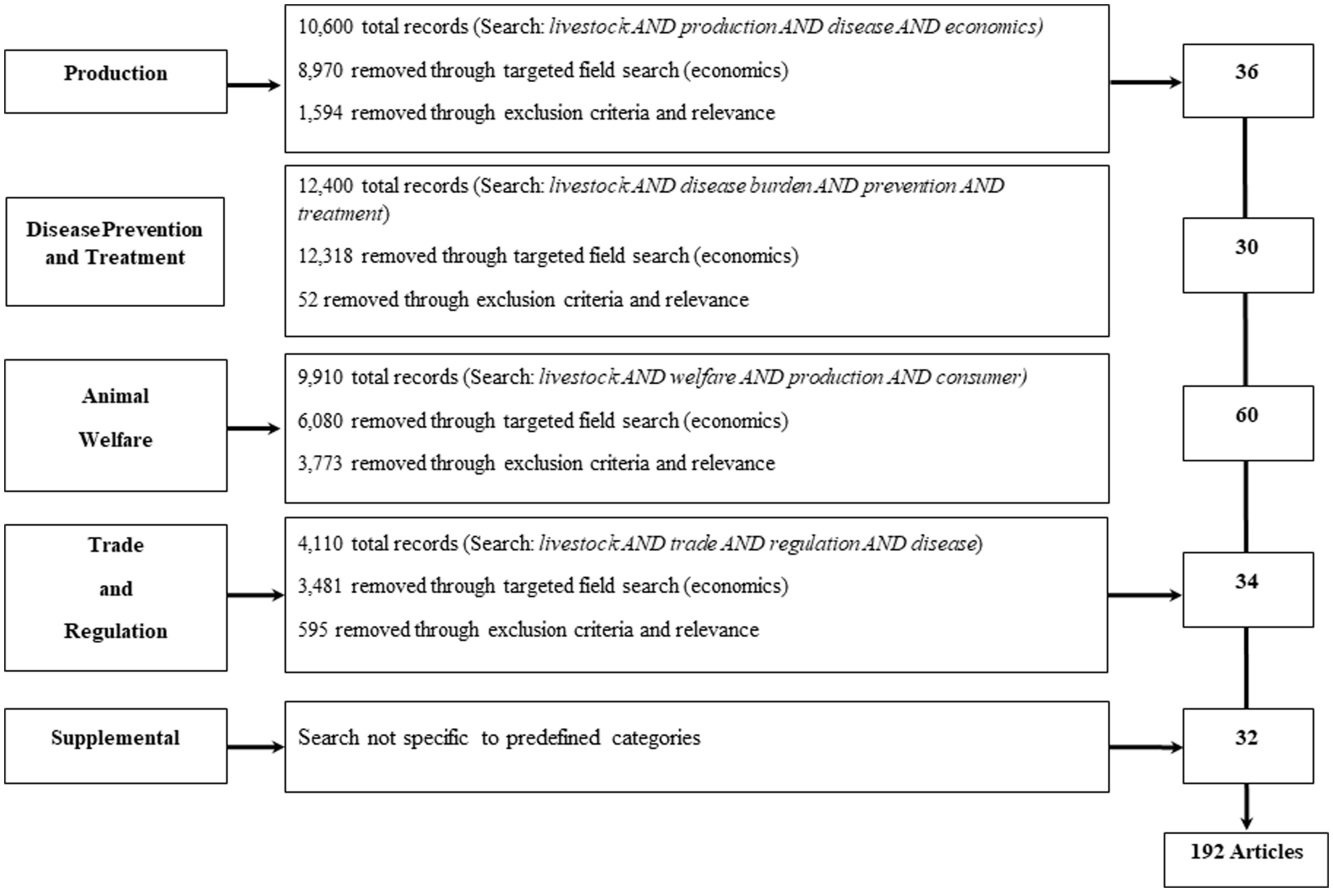
Article selection for the study’s scoping literature review.

We accomplished our objective through a scoping search of peer-reviewed literature across EconLit, AgEcon, and Google Scholar databases focusing on the major terrestrial livestock species (beef and dairy cattle, pigs, poultry, and small ruminants). We do not include time exclusion criteria in order to capture seminal research on animal disease economics but give more attention to current research on the impacts of animal disease. Also included is a search of published reports by relevant organizations such as the Food and Agriculture Organization (FAO), World Health Organization, World Organization for Animal Health (WOAH, founded as OIE), and the World Bank. This review excludes books [e.g., (29–33)]. Our initial search resulted in 184 articles and reports, and then was supplemented with eight articles from the authors. In total, 192 relevant articles were available for review across the economic categories (production, disease prevention and treatment, animal welfare, and trade and regulation; see Figure 1). The coverage across categories resulted in 19% for production, 16% for disease prevention and treatment, 30% for animal welfare, and 18% for trade and regulation. Table 1 provides additional details on the inclusion and exclusion criteria of the reviewed literature.

**Table 1:** Inclusion and exclusion criteria of the reviewed literature.

	Inclusion	Exclusion
Focus	Studies or reports on themes of production, disease prevention and treatment, animal welfare, and trade and regulation.	
Animal Group	Terrestrial	Non-Terrestrial
Search	Restricted to peer-reviewed papers from established journals and papers/reports published within an organization (ex. FAO, WHO, World Bank, etc.)	Non peer reviewed; Non reputable organization related reports
Language	English	Non-English
Context	Global with no limit	None
Year	All years	None

### Production

This section begins with a review of the costs of selected livestock diseases. Next, spillovers of livestock disease to humans and the environment are discussed and it concludes with climate change impacts on production environments and disease transmission within these environments.

A direct economic impact of clinical and subclinical livestock disease conditions is the loss of, or reduced efficiency of, production. Lost production affects food access, wealth, and income. The magnitude of the economic burden will depend on production conditions and market circumstances. If a local farm economy is diversified with alternative income opportunities, it may be more resilient, and the burden reduced. Conversely, if the local economy is dependent on one or a few vulnerable commodities, the economy may be less resilient, and the burden may be more severe and local food security impaired. The consequences of reduced productivity of animals and diseases can have lasting effects on livestock output in several “hidden” ways (such as longer reproduction cycles leading to fewer offspring) which often exceed the losses associated with visible illness (34). Examples of health problems in livestock include foot disorders (lameness), ketosis, mastitis, and fertility, which may also correspond to transboundary diseases of global importance, including lumpy skin disease, sheeppox, goatpox, and foot-and-mouth disease (FMD).

Foot disorders are reported to be a perennial problem in dairy cattle, due to their high incidence, severity, and duration. The economic consequences of foot disorders are expressed through losses in milk production, prolonged calving intervals, excessive culling, additional veterinarian visit and treatments, and as well as losses in labor for the trimmer and farmer. Evidence of total costs of foot disorders on a Dutch farm with 65 cows were $4,899 per year ($75 per cow), ranging from $3,217 to $7,001 (35). A study in Spanish dairy cows by Charfeddine and Pérez-Cabal (36) noted the phenotypic association between the severity of claws disorders and production, fertility, and performance. The authors considered three common claw disorders: dermatitis, sole ulcer, and white line disease and found the presence of sole ulcer or white line disease was associated with reduced milk production mostly in cows in second or later lactations. Further, severe sole ulcer or white line disease resulted in double the milk losses when compared to a mild condition (36). Cows with dermatitis, sole ulcer, and white line disease resulted in annual costs of $10.8, $50.9, and $43.2 per affected cow, respectively (36). Milk losses, longer calving intervals, and premature culling contributed to more than half of the additional expenditures (36). Lameness is a prominent issue in the dairy industry (37). Adams et al. (38) estimated the prevalence of lameness in United States herds as 10% in 2014, while previous literature has reported lameness prevalence to reach as high as 55% in northeastern United States herds in 2008 (39). Lameness, along with mastitis and fertility, are identified by United Kingdom dairy producers as the top three major health concerns in their herds (40).

The costs of ketosis, both clinical and subclinical, are manifested though lower milk production and reproductive performance, an increased culling of cows and other disorders (41, 42). In a study carried out in a typical Dutch dairy context, Steeneveld et al. (42) noted differences in annual net cash flows of farms in the no ketosis scenario (i.e., no risk) and the base scenario (i.e., 1% probability of clinical ketosis and 11% probability of subclinical ketosis). They report the average herd level costs of ketosis were €3,613 per year for the base scenario and €7,371 per year for the high-risk scenario [i.e., two times the clinical and subclinical ketosis probabilities; (42)].

Poxvirus diseases, mainly Lumpy skin disease, sheeppox, and goatpox, are highly contagious and can potentially cause significant losses to livestock producers through morbidity, mortality, control measures, and reduced trade (43). Infected livestock may show signs of reduced weight, reduced milk production, depression, lethargy, and fever, and, in severe cases, death. Additionally, Lumpy skin disease reduces hide quality, while sheeppox and goatpox can decrease the production of cashmere and wool. In a study conducted among backyard and transhumance producers in northeast Nigeria, producers sold cattle, sheep, and goats for 47, 58, and 57%, respectively, less than would have been sold if the animal was healthy. Limon et al. (43) also reported a 65% drop in milk production of clinically affected cows and 35% drop after they recovered. Cattle and sheep and goats lost a median of 10 and 15% of their live weight, respectively. Depending on the impacted species and production system, economic losses at the farm level range from US$10 to US$6,340 (43).

The impact of FMD has been extensively explored post-outbreak or anecdotally, but poorly characterized in endemic areas (44). An analysis was conducted to ascertain the impact on 218 lactating cattle during a 29-day FMD outbreak on milk yields. At the herd level, yields decreased from an average of 20 to 13 kg (decline of 35%) per cow per day, with recovery taking place approximately 2 months after the end of the outbreak (44). In another East African study, a 2008–2018 retrospective analysis of bovine exposure to FMD in endemic regions revealed suppressed milk and reproductive performance (45). Other considerations between endemic disease and long-term animal productivity include calving intervals and lameness.

Livestock diseases cause losses to production systems through morbidity, mortality, and prevention and control costs. While diseases usually manifest themselves as visible illnesses, they can impact productivity in a plethora of hidden ways including longer production cycles and low population growth.

### Effects of climate change on livestock and livestock diseases

Climate change may have substantial effects on the epidemiology of infectious animal diseases (46) and directly relates to production environments and subsequent impacts. Even though there might be some positive benefits of climate change on animal health, of particular interest to this review are the negative effects which manifest themselves as increased costs to livestock production. These may happen through a number of ways including increase in heat-related diseases and stress, extreme weather events, adaptation of animal production systems to new environments, and emergence or re-emergence of infectious diseases critically dependent on environmental and climatic conditions (47). These processes act by affecting the biology of the hosts, pathogens, vectors, and/or through creating environmental conditions that increase their development and contact (46). Ultimately, climate change induced impacts to animal health and well-being directly and indirectly affects livestock value chains and have welfare implications on the society at large.

Environmental temperature affects the host’s physiology, and hence, ability to respond to infection (46). For most farm animals, temperatures between 10 and 30°C are considered optimal, and higher than optimal temperatures result in reduced feed intake, milk production, reproductive performance, wool production, animal health and welfare (48, 49). Some more specific examples include a 3–5% decline in feed intake for goats, pigs, and chickens for each unit increase in temperature above optimal temperatures [National Research Council (50)]. Pigs in particular, are susceptible to heat stress when subjected to excessively high temperatures (47). In Chinese Taipei, heat stress is considered a major problem in the dairy sector. Heat stress weakens the signs of estrus, prolongs the cycle, and increases fetal death rate (47).

Temperature and moisture greatly influence the development rates, persistence, and geographical range of pathogens and vectors; hence, the transmission dynamics of vector borne diseases (46). High temperatures are generally associated with increased metabolic rates in arthropods leading to an increase in their feeding, reproduction, and maturation (51). Temperature is also an important determinant of key epidemiological factors like infection rates and dissemination patterns of pathogens in the vector. Higher temperatures shorten the incubation period of pathogens by increasing their replication rates in vectors. Temperature and humidity also influence the duration of survival of pathogens which spend part of their life cycle outside the host (52). Vectors like nematodes, mosquitoes, ticks, and flies, which are responsible for diseases affecting animals like sheep, goats, cattle, and horses, have developmental stages that are influenced by climatic conditions (47).

Climate change influences the geographical range of vectors, hosts, and pathogens. Evidence for that has been found on, for example, *Culicoides imicola* which transmits the bluetongue virus (53). Temperature and moisture frequently impose limits on the geographic range and distribution of vectors and parasites (49). In East and Southern Africa, for example, vector distribution is often limited by high mortalities during low winter temperatures and slow population recovery rates during warmer seasons. As the globe gets warmer, cooler regions, which were previously inhabitable for certain vectors, may experience increases in populations while warmer regions could remain permissive for vectors if there is also increased precipitation or humidity (49). Climate change may alter the rate at which parasites develop, resulting in an increase in some instances in the number of generations and a subsequent extension of their temporal and geographic range. The New World screwworm (*Cochliomyia hominivorax*), which is a disease that already affects animals in South America, is one such example of a disease whose geographic and temporal distribution could be changed. The spread of screwworm has been shown to be strongly correlated with amount of precipitation and temperature in Brazil (54). In some regions of Brazil, there is significant seasonal variation in the prevalence of animals with screwworm larvae, with the summer seeing the highest incidence followed by spring, winter, and autumn (54).

Climate change is predicted to alter the temporal and geographical distribution of infectious diseases in South America’s endemic regions and their introduction to disease-free areas. This includes vector borne diseases like bluetongue, West Nile fever, vesicular stomatitis, and New World screwworm (54). Despite there being historical records of bluetongue outbreaks in Europe, the recurrent introductions since 1998 have been startling. Six strains of bluetongue virus have been identified across 12 countries and have been found to occur about 800 km further north than previously reported (53). The spread into new areas of bluetongue, *Culicoides imicola* (an indigenous European midges), screwworm, tickborne diseases in Europe, South America, and Africa is mainly attributed to climate change (54). In Africa, El Niño/Southern Oscillation (ENSO) has been linked to mosquito-borne and biting midges disease (55–59). Predictions have also shown that the geographical range of some ticks (e.g., *Rhipicephalus Appendiculatus*) will likely change in some parts of Africa (46, 60). Outbreaks of bovine ephemeral (1996) and dengue fever (2001) in the Chinese Taipei were found to be linked to episodes of typhoons (47). Climate induced migration of birds may alter the geographical distribution of diseases such as Highly pathogenic Avian Influenza (HPAI) and West Nile virus (47).

Climate change is predicted to increase the frequency of droughts and floods in some parts of the world (e.g., South America and Eastern Africa) which could lead to increased movement of pastoral communities with profound effects on vector-host contact rates; hence, the spread of animal diseases (46, 54). Such movements can take producers and their animals further from key services and expose them to additional vectors and pathogens (46). Prolonged drought could also lead to aggregation of livestock production in resource abundant areas (mainly pasture and water) creating conditions conducive for the development of pathogens and vectors and their increased contact with hosts (46). The rate of contact between livestock and wildlife is also expected to increase in transhumant production systems, exacerbating the risk of spread of diseases across species, and the emergence of novel diseases (54). For example, droughts (1993–1997) in East Africa forced pastoralists to graze their cattle in wildlife areas, resulting in infections of mild lineage of rinderpest in both cattle and wildlife, devastating certain populations (49, 61). Climate-driven agricultural land use changes and biodiversity loss could expose livestock to novel pathogens. Most infectious diseases such as avian influenza, brucellosis, Newcastle disease, rabies, tuberculosis, and parasitic diseases, share wild and domestic susceptible species. Biodiversity loss is likely to lead to the emergence of novel diseases as vectors seek new hosts (54).

Although much of the previous literature has focused on the negative impacts of livestock and wildlife diseases when discussing climate change, positive benefits have also been mentioned. Moore and Messina (62) indicate that changing temperatures in different elevations in Kenya can alter vegetation structures, which impacts the soil moisture and temperature. Increases in soil temperatures and reduced soil moisture will have a detrimental impact on tsetse fly larvae and adult flies. In similar articles by McDermott et al. (63) and Lord et al. (64), they evaluate areas in Africa that could see a reduction in tsetse fly populations due to increasing temperatures and changing environmental conditions.

Climate change may affect animal health through (1) increasing the frequency and severity of climate events and associated diseases like heat stress, (2) adaptation of livestock systems to new environments, (3) promoting the emergence of novel pathogens, and (4) creating environmental conditions that increase contact among pathogens, vectors, and hosts. A significant number of studies discuss the negative impacts of climate change on animal health, but there are positive impacts as well.

### Zoonosis and other spillovers

Zoonoses are an important consideration within production environments. There are relevant diseases of dairy cattle involving pathogens transmissible from cows to humans. Eradication campaigns and the adoption of pasteurization nearly put under control such dairy diseases like brucellosis, tuberculosis, and Q-fever. In recent time, there has been few outbreaks of foodborne diseases due to the illicit consumption of raw milk. The cattle industry is currently confronted with a plethora of important public health issues like bovine spongiform encephalopathy (with variant Creutzfeldt-Jacob disease) and antibiotic use and its associated microbial resistance in humans. Environmental or ecosystem health presents additional indirect links between animals and people (65). Johne’s disease in cattle and Crohn’s disease in humans have similar pathology and this association is a developing frontier of research by veterinary and human medical practitioners.

The use of antimicrobial drugs applies selective pressure on bacteria, which can result in some bacterial strains developing antimicrobial resistance to certain drugs. There is a concern that microbial resistance could possibly be transferred from animals to people via zoonotic bacteria. More likely is the consumption of nonpathogenic bacteria, which in turn pass resistance to human pathogens (66). This spillover effect is becoming an increasing concern for human health, animal health, and the environment.

Disease spillover remains an intricate and concerning public health issue. While the advent of pasteurization helped eliminate the bulk of zoonotic concerns, issues pertaining to the association of diseases in animals and humans, and the possibility of microbial resistance transfer from animals to people are of increasing concern.

### Animal disease prevention and treatment

The increased focus on livestock production for promoting food security has called greater attention to issues surrounding food safety, moving beyond a primary focus on productivity. While consumption of animal sourced foods provides important micro and macronutrients for physical and cognitive development (67), there exists a risk of disease transmission during consumption of contaminated foods (68). Evaluation of the impact of foodborne disease has found it to be comparable to the burden of the “big three” diseases, namely HIV/AIDS, malaria, and tuberculosis, with children under five bearing 40% of this burden in low-income areas and total burden being measured at 33 million Disability Adjusted Life Years (69). Poor sanitation and hygiene in both commercial and household production environments increase risks of zoonosis (70, 71). Production intensification and urbanization further create environments where human and livestock populations are geographically concentrated and have higher potential for zoonotic transmission (1, 72). Underdeveloped areas typically lack resources and infrastructure providing sanitary and hygienic handling of livestock during production (68), which acts as an additional constraint in meeting sanitary standards for trade market access (73). Livestock disease prevention and treatment is an important production practice for mitigating negative impacts on both production and human health.

Livestock disease prevention and treatment is costly to producers. Investment in disease mitigation is typically evaluated in profit-maximizing or expenditure-loss frontier frameworks, and more generally, cost and benefit frameworks, which incorporate profits as part of benefits (20, 74–78). However, producer-level disease control decision making may not consider larger issues relating to external impacts on human health and other production systems. Absent of any disease control regulation, profit-maximizing conditions reveal that producers are not expected to eradicate all disease and will do so only when individual private benefits exceed individual costs (79).

Public policy is used to address disease prevention and treatment areas that producers do not find optimal to participate in but can negatively impact parties not immediately associated with production (80). These areas broadly relate to prevention and provision of services and include systematic vaccination and disease vector control, surveillance, diagnostics, and livestock quarantine measures, drug quality control, food and hygiene inspection, and veterinary research and extension (81). Public policy also addresses issues relating to overuse of drugs, such as antibiotics, that have production-enhancing effects but carry public health and environmental risks in the form of regulation requiring veterinary oversight for administration (82–84). Increasing fiscal deficits accounted for partly by public expenditure on disease prevention and treatment, which has been effective in lowering disease incidence, have resulted in a need to reconsider policy design and create incentive for greater private involvement (79). There is also a need to address the researcher-government relationship and the transfer of information between both parties for promoting effective policy outcomes, particularly in Africa and Asia (85).

Whether allocated through public expenditures or private markets, vaccines provide efficient means of preventing the occurrence and transmission of animal disease (86). A smallholder farmer’s decision to vaccinate livestock may not be as simple as evaluating the costs and benefits of vaccination. Adoption of vaccines can be an issue of their willingness to pay, delivery constraints, or personal beliefs and characteristics, as well as issues surrounding access and affordability (87). Evaluation of vaccination decisions among poultry farmers in Kenya against Newcastle disease found mean flock sizes increased by one bird when vaccinating and using parasiticidal treatment compared to only using parasiticidal treatment (88). Smallholder farming poultry production contributes significantly to food security, household and village livelihood, and gender equality (89) as flocks are typically managed by women within the household (88).

Local collective action supporting livestock production programs also supports vaccination programs (90). It has been found that social and cultural belief systems can influence vaccine adoption instead of household and individual factors such as income, education, age, and gender (91). In a sample of low-income Indian farmers, almost half of the sample had knowledge on how vaccines worked as a causal solution to disease, and it is expected that adoption rates improve as knowledge transfer activities take place (87), which is a consistent result across countries as dissemination of vaccine information is important for adoption (92). Vaccination decisions are also influenced by information on disease through early rapid-diagnostic testing results of foot and mouth disease, where owning larger herds is positively associated with a greater willingness to pay for early testing for informing vaccination decisions (93). It has also been found that there exists a higher willingness to pay for vaccines preventing full cattle breakdown from disease than vaccines that reduce the severity of the breakdown as it relates to bovine tuberculosis (94).

Cost of livestock vaccination is an important barrier to adoption. A veterinary intervention providing East Coast fever vaccination in sub-Saharan Africa found there to be uniform knowledge across socio-economic classes regarding vaccine benefits, but that the proportion of vaccinated cattle in herds is larger for wealthy producers compared to lower-income producers (95). This same study conducted by Homewood et al. (95) also found there to be a 50% price premium for vaccinated steers and bulls sold at market, but that cows and replacement heifers were less likely to be vaccinated due to being used for household production purposes.

On the global level, vaccination and vaccine delivery, control of livestock movement, surveillance and diagnostic testing, and systematic culling procedures contributes to the costs of transboundary disease (96), with other indirect costs including foregone revenue and secondary-industry impacts. Indirect costs of FMD prevention, control, and treatment are borne by the public and private sectors. It is estimated that 2.4 billion doses of FMD vaccines are administered annually worldwide, with a cost per vaccine between USD $0.4–3, resulting in partial indirect control costs of approximately USD $94–705 billion. Indirect cost of control also results when trade takes place between FMD-free areas. When Indonesia was an FMD-free area, it was importing large quantities of livestock from FMD-free areas. The higher price Indonesia pays is considered a risk reduction cost of not importing the FMD virus (96). Control costs required to become and stay FMD-free are significant, but benefit the aggregate economy. In Zimbabwe during 2003, it was estimated that for every Z$ 1 disinvestment in FMD control resulted in losses of Z$ 5 in terms of FMD impact on production and trade (97). In 2007, Zimbabwe was no longer able to export to the EU, foregoing historical annual trade revenues of USD $50 million (98). Control measures also have potential impact on secondary industries, where for example, the UK suffered a USD $4–5 billion loss in tourism revenue during their FMD outbreak in 2001 (99).

There is a need for accurate, cost-effective diagnostic tests for early detection of diseases in livestock (93, 100). The potential for negative livestock disease impacts on global markets increases when early detection resources are not sufficient or not available. Currently, laboratory tests for early detection of diseases are costly and time-consuming. Tools that can predict diseases incidences, including the exact livestock populations, guide diagnostics, offer treatment options, and predict the likely impacts are necessary. Novel biosensors provide significant benefits in monitoring animal health through the analysis of the animal’s environment. They are useful for early disease detection and isolation and also for monitoring of reproductive cycles (100). Nanobiosensors also lower production costs by enabling multiplexing of the bioassays on-site, thereby eliminating the need for the transportation of biological samples to laboratories.

Extant literature mentions significant advances in detection technology. Biosensors for the detection of some key diseases of economic importance have been developed. Some of these include biosensors for the diagnosis of the BHV-1 (Bovine Herpes Virus-1) viral protein, the major viral pathogen of bovine respiratory disease, FMD virus, and for the detection of the H7 and H78 strains of avian influenza (101–104). Neitzel et al. (105) have developed an indirect on-line sensor system based on the automated California Mastitis Test in milk.

Failures in disease prevention must also be recognized. For example, digital dermatitis went from being largely unknown to endemic status in most dairy herds in North America (66). Additionally, salmonellosis, leukosis, and calf diarrhea are examples in which little progress has been made, or where prevention has even regressed. The prevalence of most common diseases of economic importance has remained unchanged despite improvements in humans’ animal husbandry skills (66).

Disease prevention and control in livestock is key for mitigating negative effects on production and human health. Since diseases present themselves as negative externalities, public policy is used in disease spaces that producers may not find optimal. To date, vaccination provides efficient means of mitigating disease occurrence and transmission, and its adoption across the globe is influenced by a plethora of economic and socio-cultural factors.

### Animal welfare

Farm animal welfare (FAW) is receiving greater media and empirical attention, especially in the developed world, particularly the European Union (EU) and the United States (U.S.) (106–111). Important to highlight at this juncture is that while FAW is no longer a peripheral issue, it also not the main determinant of food purchase behavior, particularly in the United States (110). Building from the Brambell Report, and the resultant growth of ethology, there has been a surge in public awareness of FAW issues and an associated increase in animal welfare research and teaching activities (112). Goddard et al. (113), Stott et al. (114), Toma et al. (115), and Vosough et al. (116) have contributed to research spanning consumer perception of animal welfare and disease eradication across Europe. The recent European experience with animal epidemics such as bovine spongiform encephalopathy, FMD and avian influenza, and the subsequent culling of animals has also added to the growing FAW debate (117). In 2002, WOAH members voted to create international standards for animal welfare, with the initial guidelines adopted in 2005 (118, 119).

While the main FAW concerns differ across livestock species, production systems, and geography, they range from the more general like narrowly confined husbandry systems rampant in poultry, pigs, and cattle to the rather difficult and costly to implement like stopping the separation of calves from cows. Table 2 presents some of the main livestock welfare issues.

**Table 2:** Common livestock welfare issues.

Species	Issues/Concerns
Laying hens	Small battery cages, osteoporosis and beak trimming, forced molting.
Broilers	Lameness, footpad lesions (120) and hunger (feeding practices) (112), high stocking density, heat stress, and microbial contact dermatitis (121) and injury during mating for broiler breeders
Pigs	Living space (i.e., gestation stalls) and confinement conditions, castration, euthanasia, antibiotic practices, aggression, lack of stimuli
Dairy cows	Lameness, dehorning and disbudding practices, tail docking, low body condition score, treatment of bull calves, access to pasture, calf separation
Beef cows	Dehorning practices, branding, castration, transportation, heat stress, pathogen contamination of manure/mud, slaughter practices, acidosis, respiratory disease, stockmanship

Adapted from (112).

Whether it is responding to market signals or legal instruments, the main rationale for producers to respond to FAW concerns is maintaining the social license to produce (110). Husbandry methods that cause pain are constantly being reviewed and banned, not only for the welfare of animals, but also for economic reasons that healthy, happy animals perform better (122). While FAW changes in Europe were largely through a legislative approach, food retailers and food producer groups have been the leading drivers in the United States (112).

A plethora of measures have been put in place in both the EU and the United States to regulate farm animal husbandry activities and ensure that FAW considerations are part of the livestock production equation. In the United Kingdom, the Agricultural Act of 1968 provided the first legal basis for farm animal protection, leading to more explicit laws including the banning of gestation stalls in 1999 (119). Notable similar laws to ban stalls were also enacted in Sweden (1988), New Zealand (2015), Florida (effective 2008), and California (effective 2015). In 2007, Smithfield, the largest pork producer in the United States, announced they were transitioning away from gestation stalls to group housing on all its premises, including its contract growers (112). In 2021, Smithfield was sued by the Humane Society of the United States (HSUS) for misleading customers. HSUS claims Smithfield did not eliminate gestation stalls, but simply reduced the amount of time sows spend locked in the cages (123). Gestation stalls are commonly used in piggeries to increase production efficiency and improve welfare by preventing mixing of animals; thus, limiting disease spread and fighting. However, animal welfare proponents are mostly critical of the system’s effect on limiting the animal from exhibiting natural behaviors like moving around (124).

Dairy and beef production in the developed world has been commonly associated with husbandry methods warranting public FAW concerns. In recent years in the United States, undercover videos have periodically been released of poor cattle conditions and abuse on dairy farms, which focuses public attention on dairy cattle welfare issues (125). Yielding to public scrutiny and legislative pressure, certain practices have been earmarked to be phased out or banned due to associated undesirable animal welfare impacts. For instance, as of January 1, 2010, tail docking was banned in California, USA’s largest milk producing state (125). Farmers Assuring Responsible Management, a voluntary program of United States dairy farm organizations was created to champion cattle welfare issues, and in 2015, the National Milk Producers Federation announced a nation-wide end to tail docking effective December 31, 2016 (125). In addition to this producer program, various animal welfare-related groups have created their own programs that have certification and labeling for marketing purposes (e.g., Humane Farm Animal Care). Tail docking is now a rarity in the United States. Tail docking was once perceived to lower the risk of zoonotic disease leptospirosis although this belief is now defunct (126), and neither did studies establish the purported effect of docking on udder infection or mastitis (127–132).

Dehorning and disbudding are characteristics of intensive cattle production. Dehorning is aimed at limiting injuries from animals and economizing on space requirements (133). Due to public FAW outcry, there is shift toward reducing the pain associated with the process. A study in Alberta, Canada revealed that while dehorning and branding remains common in cattle ranching, ranchers and farmers are slowly moving toward practices that induce less pain, mainly through the use of caustic paste for disbudding (122, 133). Potential also exists for gene-editing technology to produced polled cattle, although uncertainty remains on consumers perceptions for gene-edited food products. Genetic dehorning eliminates the need for a painful dehorning, a process resented by animal welfare activists (134).

Separation of cows from calves soon after parturition, and the separate housing of calves during the milk-feeding period are common practices in the dairy industry (135). Abrupt weaning is still a persistent and common practice and those who practice it claims that it reduces emotional distress for the animals and promotes calf health. However, advocates of FAW argues it is a stressful process that affects the calves physical and physiological development (132, 135). A recent study conducted in Canada reveals that some producers are adopting weaning methods with low stress [e.g., fence-line weaning and two-stage weaning; (135)].

Zero grazing is increasingly becoming contentious in countries where total confinement of animals has become a norm (132). In 1998, Sweden enacted a law essentially putting an end to zero grazing of dairy cows (119). Access to pasture is valued by some people because it also offers enough space and fresh air to animals. In the United States, about 39% of dairy farms use tie stalls, and the majority of lactating cows are kept in total confinement (136). Some of the reasons hampering availing pasture to dairy cattle include difficulties in incorporating pasture into modern farms, pasture shortages, low veld quality, and fears of lower milk production from pasture access (132).

Significant strides have been made as far as poultry FAW issues are concerned. There has been a widespread ban on the on the use of conventional battery cages for hens notably in Sweden (1998), EU (2012), New Zealand (2022), and California (2015) (119). In the United States, the United Egg Producers (UEP) is one of the first groups to be at the forefront of sweeping changes related to poultry husbandry in response to public poultry welfare concerns. Mainly, an increase in cage space, to 67–87 in.^2^ per hen from the current industry standard of 48–54 in.^2^ per hen, which was implemented over a seven-year span by producers to buffer economic impacts. These guidelines also include standards for lighting, air quality, beak trimming, handling, and on-farm euthanasia. Additionally, in 2006, food withdrawal to induce molting in hens was banned, followed by inclusion of cage-free production standards by 2008 (112). To implement these guidelines, a third party auditing program was developed and allowed qualifying producers to display a logo on their egg cartons showing that they are UEP certified.

In emerging and developing countries, the issue of FAW is also gaining momentum due to global export requirements as well as domestic concerns from the burgeoning middle class (106, 137). This momentum will likely be augmented by international corporations in the food business that define global supply chain requirements and ultimately shape livestock production practices and consumption in these countries (106, 138). However, there are still several factors that can potentially slow down changes aimed at improving husbandry practices in both emerging and developing countries. In emerging countries like Brazil, in the quest to meet domestic and export market meat demand, there has been a deliberate transition toward the adoption of the very intensive husbandry practices that are at the center of FAW outcries in the developed world. These controversial husbandry practices place them in an especially vulnerable position, and it’s a matter of time that they too attract similar scrutiny (119).

Given the prevalence of food insecurity and poverty in developing countries, this means that FAW receives low priority. Limited access to animal handling technologies, the relative absence of societal pressures for improved welfare, and substandard handling facilities contribute to its low priority (139). For cattle production, the dominant communal production systems mean that cattle are reared for several purposes (i.e., meat, milk, draft, and traditional ceremonies). In these systems, nutritional deficiencies are commonplace due to deteriorating rangelands, especially in winter or dry seasons. In addition, most developing countries have dysfunctional or non-existent animal health systems (139).

Important to note is that any FAW investment or adjustment comes at a cost (110). Paramount to the sustainability of efforts to address FAW issues therefore is the existence of a willingness to pay (WTP) for such improvements from consumers, or at least some form of subsidies. There has been an increase over the past two decades in the number of citizens and consumers with deep regard for FAW and professed their unwillingness to buy products that did not meet their FAW concerns (140). Consumers associate FAW not only with higher human health benefits but also consider food produced under FAW friendly conditions to be of higher quality, tastier, more hygienic, safer, acceptable, true to type, eco-friendly, and traditional (141–145).

A meta-analysis conducted using 23 WTP studies mostly from the OECD countries revealed that WTP for improved FAW is on average approximately 15% above base price, and comparatively higher than that of the United States (106). Results of surveys conducted in Europe show an increase from 34 to 57% between 2006 and 2015 of the proportion of citizens who assign some importance to the protection of farmed animals (140). Various studies have reported a similar trend world-wide; EU (146–149), United States (125, 150–152), Canada (153, 154), Latin America (137, 155–157), Asia (158), and Australia (159). Given that there is a correlation between income levels and demand for FAW, this issue is likely to continue being of interest for the unforeseeable future as more and more countries emerge from poverty (160).

In some policy circles, individuals argue that FAW is overrated and just a fashionable cause (106). Older, wealthier, and female consumers tend to be more concerned with FAW when purchasing food (110). Firstly, duality exists between ordinary citizens and consumers (i.e., not all consumer concerns are reflected in their purchase behavior). Empirical studies reveal mismatch between the results of self-reported public concerns about FAW and the WTP for products that comply with FAW standards, the attitude-behavior gap (106, 140, 161). Effective demand for FAW is only reflected in the food choices and purchases of consumers while citizens only partake in activism, political processes, and formation of public opinion in which their stated desire for FAW change is victim to social desirability bias [i.e., respondents give answers to questions that they believe will make them look good to others; (110, 162)].

In Germany, over two-thirds of consumers expressed disdain with existing FAW unfriendly husbandry practices (107), but rather paradoxically is the small niche of organic meat (2% market share) (163). A small proportion of United States consumers care a great deal about FAW to the extent that it influences their food purchase behavior (110). Lister et al. (164) revealed low importance of FAW among United States consumers across several foods: ground beef (5.2%), beefsteak (4.6%), chicken breast (4.1%), and milk products (4.8%). Conversely, the relative importance of price was consistently around 20%, demonstrating that price remains by far the most important drive of purchase behavior among United States consumers (110).

Besides price, FAW is confounded by other factors like food safety which take precedence over all others when it is present or perceived (110). Harper and Henson (165) reported that in the UK, Ireland, France, Germany, and Italy consumers prioritize food safety, health, and quality over FAW concerns. Consumers considered FAW as an indicator of other attributes associated with human health and safety (166). FAW is therefore more likely to be valued by consumers when it is part of a broader basket of private values.

The other challenge with the market of FAW is its public good characteristics which creates a positive consumption externality, and a consequent free-rider incentive. As far as FAW is concerned, there is non-excludability and non-rivalry in consumption (106, 163). Market demand for FAW does not truly reflect preferences. The burden of the externality is borne only by a segment of consumers (167). The free-rider incentive can be lessened by a perceived increase in the private value (e.g., nutrition and taste). We explore the implications of consumer preference below.

### Consumer preferences

Willingness to pay (WTP) for improved health management inputs captures producer demand for these improvements but can also be applied to consumer demand for animal products. Increasingly, consumer preferences for characteristics beyond disease or quality, such as for animal welfare, may also be tied to demand, especially in Europe and the United States (106–111). While animal welfare is not a fringe issue, it also is not a main driver of food demand, particularly in the United States (110), and instead helps with issue framing to indirectly affect demand (112).

Willingness to pay for health inputs has been extensively explored in animal health economics. For endemic diseases, WTP captures potential markets for vaccine manufacturers or potential for public investment. FMD WTP has been explored in Africa and shown to be a potentially cost-effective approach whereby producer WTP either aligns or is above the price point to offer the vaccine. In these cases, when consumer demand is higher than market value, the excess in consumer demand (consumer surplus) often can translate into widespread adoption. During the last vaccination campaign, a WTP study of Rift Valley Fever (RVF) vaccine in Kenya found that WTP ranged from 17 to 67% higher than the costs incurred by government ($0.86 USD per head of cattle). However, producers appeared to be sensitive to total costs whereby producers with many cattle had lower WTP values. A study to assess WTP for a bovine tuberculosis cattle vaccine in England and Wales similarly found that WTP values were substantially higher than the expected cost of a vaccine (94). Demand for improved animal health is not limited to bovines or large investments. A WTP study for Newcastle disease vaccines found that on-farm income would likely be sufficient to cover vaccination costs and that low-income households valued the vaccines more (168).

WTP studies and those assessing uptake of animal health inputs may also be applied to exotic and zoonotic diseases as they often incorporate the risk of disease incidence into the evaluation. In the same study that evaluated WTP for FMD vaccines in an endemic region of East Africa (169), the study found that WTP was influenced by disease risk perceptions, such that a more spatially and temporally immediate outbreak may be met with a higher WTP for prevention and control. Kairu-Wanyoike et al. (170) found in Kenya that the WTP for a contagious bovine pleuropneumonia (CBPP) vaccine was constrained by access to information on disease risk. An exploration of vaccine uptake for Newcastle disease in Tanzania similarly found that access to professional-level information (potentially through veterinarians) was associated with increased uptake (171).

Farm animal welfare issues have been gaining momentum mainly in the developed world where they are primarily driven by legislature, food producers, and food retailers. Though FAW is not a fringe issue, price remains the key determinant of food demand. FAW is also gaining relevance in low and middle-income countries mainly due to global supply chain requirements defined by large food corporations. Its prioritization is hampered by food insecurity, poverty, and the need to meet domestic and export demand. There exists a gap between high public FAW sentiments and relatively low levels of FAW willingness to pay.

### Trade and regulation

Within this section we explore the implications of livestock disease on global markets. We first explore the risks of transboundary livestock disease on trade and then turn to the impacts of regulatory measures, and subsequent market outcomes, on producers and consumers within respective import and export markets.

Access to and participation in efficient trade markets will increase incomes and decrease unemployment in comparison to existing levels without trade. The availability of pasture area per head of rural population in underdeveloped areas provides a comparative advantage for livestock production and export (172). Considering efficient and safe markets, areas with less-efficient production will have incentive to import livestock products from areas with a production advantage.

Trade is promoted through investment in infrastructure and supply chain organization in areas with growth in commercial enterprise and areas dependent on smallholder farming systems for livestock products. Both infrastructure and organization act as compliments for establishing and sustaining trade markets (173), with public and private sector involvement and partnership remaining equally important (174).

Average public investment from OECD countries in agriculture within sub-Saharan Africa has increased by 87.5% from USD $0.08 billion to USD $0.15 billion over the period 1980–2012 (175). While accurate and comprehensive data on private investment in developing areas is not readily available and/or accessible, an analysis of foreign direct investment in agribusiness has shown low but slowly increasing levels of investment in areas typically focused on value-added processes (176). Public programs targeting increased private investment for agricultural development, such as the Comprehensive Africa Agriculture Development Program, show the importance of private sector funding and the public-private relationship.

Trade has the potential to increase the risk of zoonotic disease and foodborne related illnesses. Compliance with regulatory safety standards and inspections, as well as public and private certification of livestock and livestock products mitigate risks of disease and illness transmission. Market inefficiencies arise when trade embargoes, which ban all export goods from one country or region, or tariffs, which decrease consumption of goods by making them more costly, are enacted in response to a livestock disease outbreak in an exporting country, or in response to a country’s failure to adhere to established safety standards and inspections.

Transboundary diseases occur alongside trade and can threaten the continuity of international trade. The FAO defines transboundary animal diseases as those that are highly contagious and easily transmissible across borders and that have negative impacts on socioeconomic and public health outcomes, ultimately placing risks on trade (34). Economic impacts of transboundary diseases include public and private costs of outbreak, as well as individual costs of disease prevention, control, and total loss. Wider market impacts due to shifts in consumer preference during an outbreak, or trade bans and restrictions in response to an outbreak, include changes in consumer and producer surplus, which can be thought of as a measurement of benefit gained from market participation, as well as costs imposed on secondary industries as a result of market impacts. Food security and nutrition in developing areas is also negatively affected by transboundary disease when substitution across animal-sourced foods is not possible. Disability-Adjusted Life Years (DALYs) can be used as a public health measurement of the burden of infectious disease in humans through loss in life years due to poor health, disability, and death (177) that may occur in transboundary disease incidents. Infectious disease accounts for 30% of global DALYs (18), with an estimated 60% of infectious diseases being zoonotic (178). While any value chain is subject to the negative impacts of transboundary disease, smallholder farming systems have greater vulnerability (179). The World Organization for Animal Health lists notifiable transboundary diseases, among them are FMD, rinderpest, African and Classical swine fever (ASF and CSF, respectively), CBPP, and RVF. Prevention, monitoring, and effective response to FMD and African and classical swine fever carries high importance as they are three of the most detrimental diseases to producer livelihood and international trade outcomes (180).

Foot and mouth disease is highly contagious in cattle and other cloven-hoofed animals, and spreads through populations during movement of infected animals and products. Access to trade markets is restricted to countries and regions free of FMD. As an example, in 1996 Uruguay was recognized as being free of FMD and gained access to valuable trading markets. Uruguay filled export quotas to the United States and received higher world prices than domestic prices, resulting in additional annual revenues estimated at USD $20 million (181). Access to Pacific Rim trade markets was estimated to provide an additional USD $90 million annually. As restriction from trade is an indirect cost of transboundary disease, Uruguay’s total additional revenue gained contributes to the full costs of FMD. Cost–benefit analysis of FMD eradication in Bolivia and Thailand found that benefits exceeded costs only if eradication allowed participation in trade markets (34). It is estimated that Latin America experienced a 4.1% decrease in meat exports attributed to their FMD outbreak in 2001 (182). During trade restrictions, export production increases domestic supply, resulting in lower domestic prices and consumers benefit from these lower prices under the assumption that products are safe and marketable, and meat substitution is not made. However, it is important to consider a country’s depopulation strategy and domestic demand elasticity when measuring welfare changes between consumers and producers. Given inelastic demand for domestic beef, if depopulation reduces excess supply enough, producer surplus increases while consumer surplus decreases during an outbreak (183). During an evaluation of the Australia’s livestock export industry, increased domestic supply of livestock products due to trade restrictions resulted in a greater-than USD $1.5 billion industry loss during a hypothetical FMD outbreak. A simulated study of a FMD outbreak in the United States found that total livestock industry losses occurred over 16 quarters until recovery, totaling between USD $2.8–4.1 billion due to impacts on trade, domestic supply, and demand (184). In another study simulating an FMD outbreak in the United States, producer sector welfare, defined as the change in producer surplus for non-quarantined livestock and the loss in sales revenue for livestock quarantined and slaughtered, declined by USD $1.4–1.8 million (185). In general, it is estimated that market prices for major beef importing and exporting countries among FMD-free areas will be up to 50% greater than domestic prices received in FMD areas (186). Other FMD case studies include control and vaccination strategies across the United Kingdom and South Vietnam (187, 188). Optimal disease response strategies minimize socioeconomic disruptions at the local and national levels and are weighed against impacts of loss in export trade, its associated value, and market restriction (189).

In the Knight-Jones and Rushton (96) review of FMD impacts, general findings across simulations and data analysis point toward FMD control programs generating positive returns to an economy; FMD-free areas suffering a 0.2–0.6% loss in GDP during a new outbreak, with Taiwan experiencing a 0.28% loss in GDP across multiple sectors during their 1997 outbreak (190); and that there exists an absence of studies exploring the full economic impacts of FMD in endemic areas with focus on indirect impacts at national levels.

African swine fever was recorded in China for the first time in 2018. It is estimated that 150–200 million pigs were infected by 2019, approximately 30% of the Chinese pig population, with disease impacts causing almost 100% mortality as there is currently no vaccine. In a simulated reduction of 9–34% of global swine production due to the Chinese outbreak, global pork prices were estimated to increase by 17 to 85% (191), having global economic impacts on consumer and producer surplus, and nutrition substitutions. In a simulated study of an ASF outbreak in Iowa, United States, which is a predominant swine production area, the outbreak would result in the loss of international markets to United States pork, with a decrease of domestic live hog prices by 40–50% due to supply surplus. The outbreak is estimated to have industry losses of USD $50 billion across all years of the outbreak due to trade restrictions and domestic price reductions (192).

Classical swine fever control mainly stems from vaccination and variable stamping-out strategies in Central and Eastern Europe countries, with most of the countries having legislation prohibiting swine imports from infected areas (193). Trade and industry costs of the 1997 outbreak of CSF in the Netherlands resulted in a 24% reduction in net cash-flow throughout densely populated livestock areas (194), and a EUR 636 million reduction in net welfare, which measures the collection of consumer, producer, and government welfare (195). Although the United States has been CSF free since 1976, a study evaluated a hypothetical CSF outbreak in the United States and estimated that full export market recovery does not occur until 14 quarters after the initial outbreak with expected total industry losses between USD $2.6–4.1 billion (196).

While trade offers opportunities for countries to exploit comparative advantages, it has the potential to increase the risk of zoonotic disease and foodborne-related illnesses. Trade facilitates the movement of animal diseases across borders which can threaten the continuity of international trade. Effective prevention and monitoring of FMD, ASF, and CSF in global livestock production systems, as well as all transboundary diseases, helps mitigate risks of trade bans and restrictions, and its subsequent effect on livelihoods, associated industries, and national/regional economies. It is important for private and public stakeholders to consider costs related to virus control, disease spread, prevention and zoning, as well as costs related to market and price shocks in the affected livestock sector and across associated sectors (197–199). Benefits to society from access to and participation in trade, and from efficient domestic markets, validate the cost of disease prevention and eradication (184).

## Conclusion

The impact of animal disease and health on markets and livelihoods is complex and is heterogeneous from region to region across the world. This review has identified selected, current knowledge surrounding the impacts of and attention to animal disease across production, disease prevention and treatment, animal welfare, and trade and regulation. The design of this study provides a broad review of some main topics within the animal health economic literature that complements other recent reviews. We recognize that this broad overview does not dive deep into mechanisms or behavioral issues for and implications of animal health in global and regional markets, as well as not fully addressing livestock-wildlife interactions, topics in diversity, equity, and inclusion, nor zoonotic diseases, which can be considered limitations of the study yet to be explored. We encourage the reader to use this review as a guide to investigate topic gaps further.

The impact of animal disease on food security and human health has focused attention on merging economics with epidemiology. Policy is not only informed by effects on supply, market price, and trade, but also informed by human health impacts. Due to the heterogeneous nature of livestock and livestock product value chains in both developed and underdeveloped areas, there exists a gap in knowledge on the distribution of animal disease burden within value chains and its effect on the wider economy. Obtaining accurate and appropriate data and institutional information is needed to assess impacts of animal disease burden segmented across all participants and economies that make up value chains. Assessing the global burden of animal disease requires a multidisciplinary approach between information, population and production systems, economic and epidemiological analysis, as well as animal health ontology and human health impacts. Work that can address the burden of animal disease would provide improved access to knowledge on the immediate and wider impacts of animal disease on industries and economies. Evaluating how animal disease burden is distributed across the value chain and its impact on the value chain and economy better informs policy and allows targeted investment from private and public organizations (13, 200).

## Author contributions

JR, KM, DP, and TM conceived the study. AK and TT carried out the literature review and drafted the preliminary manuscript. All the authors participated in reviewing, editing, reading, and approving the final draft.

## Funding

This research is performed in the framework of the Global Burden of Animal Diseases (GBADs) programme which is led by the University of Liverpool and the World Organization for Animal Health (WOAH) https://animalhealthmetrics.org/. This research is supported through the Grant Agreement Investment ID INV-005366 with the Bill & Melinda Gates Foundation and the UK Foreign, Commonwealth and Development Office (FCDO).

## Conflict of interest

The authors declare that the research was conducted in the absence of any commercial or financial relationships that could be construed as a potential conflict of interest.

The handling editor BH declared a past co-authorship with the author JR and declared a shared research network [Network for Ecohealth and One Health, NEOH–European chapter of Ecohealth International association] with the author KM.

## Publisher’s note

All claims expressed in this article are solely those of the authors and do not necessarily represent those of their affiliated organizations, or those of the publisher, the editors and the reviewers. Any product that may be evaluated in this article, or claim that may be made by its manufacturer, is not guaranteed or endorsed by the publisher.
